# Does neuroinflammation fan the flame in neurodegenerative diseases?

**DOI:** 10.1186/1750-1326-4-47

**Published:** 2009-11-16

**Authors:** Tamy C Frank-Cannon, Laura T Alto, Fiona E McAlpine, Malú G Tansey

**Affiliations:** 1 Department of Veterinary Integrative Biosciences, College of Veterinary Medicine & Biomedical Sciences, Texas A&M University, College Station, TX 77843, USA; 2 Department of Physiology, The University of Texas Southwestern Medical Center, Dallas, TX 75390, USA; 3 Cancer Research UK London Research Institute, 44 Lincolns Inn Fields, WC2A 3PX, UK; 4 Department of Physiology, Emory University School of Medicine, Atlanta, GA 30322, USA

## Abstract

While peripheral immune access to the central nervous system (CNS) is restricted and tightly controlled, the CNS is capable of dynamic immune and inflammatory responses to a variety of insults. Infections, trauma, stroke, toxins and other stimuli are capable of producing an immediate and short lived activation of the innate immune system within the CNS. This acute neuroinflammatory response includes activation of the resident immune cells (microglia) resulting in a phagocytic phenotype and the release of inflammatory mediators such as cytokines and chemokines. While an acute insult may trigger oxidative and nitrosative stress, it is typically short-lived and unlikely to be detrimental to long-term neuronal survival. In contrast, chronic neuroinflammation is a long-standing and often self-perpetuating neuroinflammatory response that persists long after an initial injury or insult. Chronic neuroinflammation includes not only long-standing activation of microglia and subsequent sustained release of inflammatory mediators, but also the resulting increased oxidative and nitrosative stress. The sustained release of inflammatory mediators works to perpetuate the inflammatory cycle, activating additional microglia, promoting their proliferation, and resulting in further release of inflammatory factors. Neurodegenerative CNS disorders, including multiple sclerosis (MS), Alzheimer's disease (AD), Parkinson's disease (PD), Huntington's disease (HD), amyotrophic lateral sclerosis (ALS), tauopathies, and age-related macular degeneration (ARMD), are associated with chronic neuroinflammation and elevated levels of several cytokines. Here we review the hallmarks of acute and chronic inflammatory responses in the CNS, the reasons why microglial activation represents a convergence point for diverse stimuli that may promote or compromise neuronal survival, and the epidemiologic, pharmacologic and genetic evidence implicating neuroinflammation in the pathophysiology of several neurodegenerative diseases.

## Overview

Once considered an immune-privileged site because of the presence of the blood brain barrier (BBB), it is now clear that while peripheral immune access to the central nervous system (CNS) is restricted and tightly controlled, the CNS is capable of dynamic immune and inflammatory responses to a variety of insults [1]. Infections, trauma, stroke, toxins and other stimuli are capable of producing an immediate and short lived activation of the innate immune system within the CNS [2,3]. This acute neuroinflammatory response includes activation of the resident immune cells (microglia) resulting in a phagocytic phenotype and the release of inflammatory mediators such as cytokines and chemokines [4]. While an acute insult may trigger oxidative and nitrosative stress, it is typically short-lived and unlikely to be detrimental to long-term neuronal survival. Therefore, it is believed that an acute neuroinflammatory response is generally beneficial to the CNS, since it tends to minimize further injury and contributes to repair of damaged tissue.

In contrast, chronic neuroinflammation is a long-standing and often self-perpetuating neuroinflammatory response that persists long after an initial injury or insult. Chronic neuroinflammation includes not only long-standing activation of microglia and subsequent sustained release of inflammatory mediators, but also results in increased oxidative and nitrosative stress [4]. The sustained release of inflammatory mediators works to perpetuate the inflammatory cycle, activating additional microglia, promoting their proliferation, and resulting in further release of inflammatory factors. Owing to the chronic and sustained nature of the inflammation, there is often compromise of the BBB which increases infiltration of peripheral macrophages into the brain parenchyma to further perpetuate the inflammation [1]. Rather than serving a protective role as does acute neuroinflammation, chronic neuroinflammation is most often detrimental and damaging to nervous tissue. Thus, whether neuroinflammation has beneficial or harmful outcomes in the brain may depend critically on the duration of the inflammatory response.

Neurodegenerative CNS disorders, including multiple sclerosis (MS), Alzheimer's disease (AD), Parkinson's disease (PD), Huntington's disease (HD), amyotrophic lateral sclerosis (ALS), tauopathies, and age-related macular degeneration (ARMD), are associated with chronic neuroinflammation and elevated levels of several cytokines [5-8]. Neuropathological and neuroradiological studies indicate that neuroinflammatory responses may begin prior to significant loss of neuronal populations in the progression of these diseases. While there is no evidence to support a role for any particular cytokine in the direct triggering of any of these neurodegenerative conditions, cytokine-driven neuroinflammation and neurotoxicity may modify disease progression in a number of these disorders. For example, inflammatory challenges might act as triggers to uncover pre-existing genetic vulnerabilities that contribute to neuronal dysfunction and death. Alternatively, viruses or bacteria might "prime" the immune system to respond aberrantly to subsequent environmental challenges. The purpose of this article is to review the evidence that microglial activation represents a convergence point for diverse external stimuli that promote neuronal dysfunction and hasten neuronal death, and the epidemiologic, pharmacologic and genetic evidence implicating neuroinflammation in the pathophysiology of several neurodegenerative diseases. If the available evidence supports a role for neuroinflammation in any of these diseases, it may be possible to alter the course of disease development in afflicted individuals with timely delivery of anti-inflammatory therapy.

## Microglia activation: convergence point for diverse stimuli that compromise neuronal survival

Microglia are the resident tissue macrophages in the central nervous system and are the principle mediators of inflammation. In the resting state, microglia display a small cell soma and numerous branching processes (a ramified morphology). In healthy brain tissue, these processes are dynamic structures that extend and retract sampling and monitoring their microenvironment (Nimmerjahn 2005, Raivich 2005). During the resting state several key surface receptors are expressed at low levels; these include the tyrosine phosphatase (CD) 45 (also known as leukocyte common antigen), CD-14, and CD11b/CD18 (Mac-1) (Kreutzberg 1996). In addition cell surface receptor-ligand pairs such as CD200R/CD200 are present to maintain neuron-glia communication in the CNS (Hock 2000, Cardona 2006).

In the presence of an activating stimulus, microglial cell-surface receptor expression is modified and the cells change from a monitoring role to one of protection and repair (reviewed in [4,9]). In addition to up-regulation of the key surface receptors mentioned above, there is up-regulation of proteins such as CD1, lymphocyte function-associated antigen 1 (LFA-1), intercellular adhesion molecule 1 (ICAM-1 or CD54), and vascular cell adhesion molecule (VCAM-1 or CD106). Activated microglia secrete a variety of inflammatory mediators including cytokines (TNF, and interleukins IL-1β and IL-6) and chemokines (macrophage inflammatory protein MIP-1α, monocyte chemoattractant protein MCP-1 and interferon (IFN) inducible protein IP-10) that promote the inflammatory state. The morphology of the cells changes from ramified to amoeboid as they take on a phagocytic role. These moderately active microglia are thought to perform beneficial functions, such as scavenging neurotoxins, removing dying cells and cellular debris, and secreting trophic factors that promote neuronal survival. Persistent activation of brain-resident microglia may increase the permeability of the BBB and promote increased infiltration of peripheral macrophages, the phenotype of which is critically determined by the CNS environment [10].

**Figure 1:**
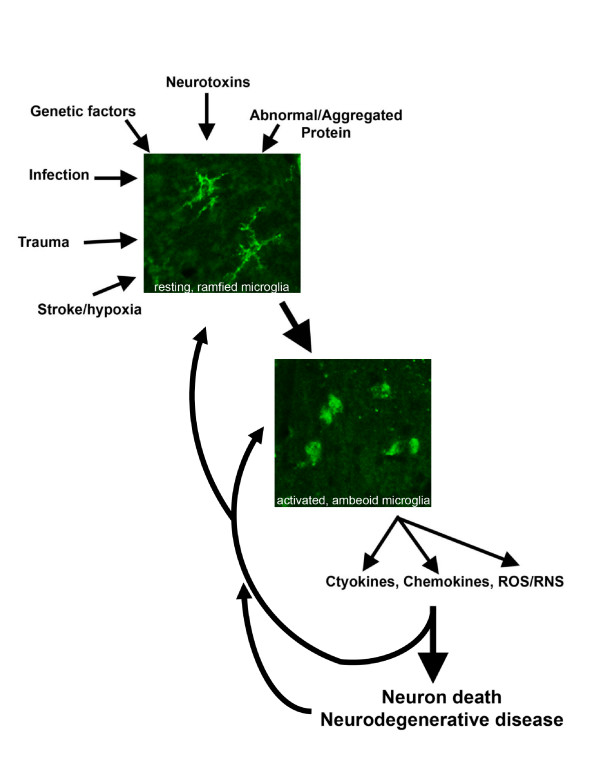
**Neuroinflammation can precede and contribute to neuronal dysfunction and degeneration**. Divergent initiating triggers directly or indirectly converge to activate microglia (stained here with an antibody against F4/80) from a ramified/resting state to an ameboid-shape/activated state, initiating a self-propelling cycle of neuroinflammation and chronic over-production of inflammatory mediators. These mediators impact susceptible neuronal populations in the CNS and contribute to their demise within the context of each neurodegenerative disorder. The progressive loss of neurons that characterizes these disorders further contributes to generation of debris and keeps microglia activated indefinitely maintaining microglia in an activated state long-term.

Microglia are the critical convergence point for the many diverse triggers that elicit an adaptive immune response (Figure 1). Stroke, hypoxia, and trauma compromise neuronal survival and indirectly trigger neuroinflammation as microglia become activated in response to the insult in an attempt to limit further injury. Infectious agents activate microglia either through damage to infected cells or direct recognition of foreign (viral or bacterial) proteins. Following exposure to neurotoxins such as the mitochondrial complex I inhibitor 1-methyl-4-phenyl-1,2,3,6-tetrahydropyridine (MPTP), the dopamine analog 6-hydroxydopamine (6-OHDA), or the pesticide paraquat, microglia become activated and primed. Microglial responses to these toxins may contribute to neuronal dysfunction and eventually hasten neurodegeneration (Czlonkowska et al., 1996; Kohutnicka et al., 1998; Liberatore et al., 1999; Dehmer et al., 2000; Vila et al., 2001). In addition, genetic mutations that give rise to increased production of toxic oligomeric, aggregated/truncated, or oxidized protein species promote sustained activation of microglia and may prime the immune system for aberrant responses to subsequent insults. Regardless of the initiating factor, all of these external or internal stimuli have the potential to trigger a self-perpetuating inflammatory response that, if left unresolved, may contribute to death of vulnerable neuronal populations.

## Evidence of neuroinflammation and cytokine involvement in neurodegenerative diseases

### Alzheimer's Disease (AD)

Although the etiology of sporadic AD in humans is unknown, mutations in Amyloid Precursor Protein (APP) or components of its processing machinery (β-secretase and γ-secretase) result in overproduction of Aβ1-40 and 1-42 peptides and are sufficient to cause disease [11-17]. Over 20 years ago, microglia were reported to localize to amyloid plaques in AD brain [18] and since then, the association between neuroinflammation and AD has been extensively investigated [7,19-24] and reviewed [19,25]. Human microglia display an activated phenotype when they surround plaques [23] that includes upregulation of Human Leukocyte Antigen-DR (HLA-DR) [18]. In addition to producing cytokines and other pro-inflammatory mediators [19,26], microglia have also been reported to exert toxicity on neurons that have been pre-exposed to low concentrations of Aβ42 via a CD14-dependent process [27]. Although it is clear that not all microglia activation is injurious to neurons, it is becoming widely accepted that a type of neurotoxic microglia phenotype has a central role in the pathophysiology of AD.

While microglia can become activated after exposure to fibrillary Aβ *in vitro *and are capable of phagocytosing it *in vitro *[28-30], it has been reported that in the presence of inflammatory cytokines or certain extracellular matrix proteins, microglia cannot phagocytize Aβ [31]. This observation has been used to support the idea that the persistence and accumulation of amyloid plaques *in vivo *may be a direct result of this inhibitory behavior exerted by neuroinflammation. Moreover, numbers of IL-1α positive (i.e., pro-inflammatory) microglia are increased in the cortical layers affected by plaque pathology in AD patients [32]. Since microglia are found in large numbers around neuritic amyloid plaques, but not diffuse in human AD patients, and in mice transgenic for mutant APP [33], it has been postulated that microglia play a role in the conversion of diffuse to neuritic senile plaques but not in the origin of diffuse plaques [34]. Specifically, post-mortem analyses of microglia density in the neocortex of three groups of nondemented individuals at different stages of senile plaque formation versus AD patients revealed that the mean density of microglia was highest in the AD group and that microglia were associated with neuritic plaques much more often than with diffuse plaques Interestingly, individuals with some neuritic plaques also had microglia within a greater proportion of their diffuse plaques than individuals who only had diffuse plaques. While these findings raise the possibility that microglial activity may promote conversion of diffuse plaques into neuritic plaques, an important caveat of this study is that this conclusion is based on correlative observations postmortem and were not derived from a dynamic *in situ *analysis of microglial activity around diffuse or neuritic plaques.

The pro-inflammatory effect of Aβ deposition in brain has been recapitulated in aged mice transgenic for a familial AD mutation of APP, in which astrocytes and microglia expressing Il-1β, IL-6 and TNF have been found surrounding amyloid plaques [35]. The bacterial endotoxin lipopolysaccharide (LPS), a powerful inducer of inflammatory responses [36], exacerbates the appearance and severity of AD pathology in the APPV717F transgenic mouse [37], the APPswe transgenic mouse [38], and the triple-transgenic (3xTgAD) mouse [39]. In addition, the Transforming Growth Factor-β (TGF-β) cytokine family increases Aβ accumulation in the cerebral blood vessels of mice transgenic for human APP and is upregulated in blood vessels of human patients with Cerebral Amyloid Angiopathy (CAA) [40]. Co-addition of TGF-β1, 2 and 3 isoforms with Aβ causes increased Aβ accumulation in organotypic hippocampal slices [41]. Together, these findings demonstrate the close association between microglia and plaque deposition and the ability of certain chronic inflammatory stimuli to exacerbate and accelerate amyloid-associated pathology. The emerging idea is that an inflamed CNS environment may influence the ability of microglia to contribute to plaque deposition rather than plaque removal, strongly suggesting that the microenvironment of the brain can influence whether microglia perform beneficial or deleterious functions in pathophysiological states.

While the role of inflammatory responses in AD are just beginning to be understood, there is little dispute that inflammatory mediators may represent both potentially useful biomarkers and targets for drug development. Cyclooxygenase 2 (COX2), a pro-inflammatory protein that is one of the targets of non-steroidal anti-inflammatory drugs (NSAIDs), and its homolog COX-1 [42], are elevated in AD brains [43]. Serum levels of the acute phase protein α1-antichymotrypsin, which is upregulated by injury, trauma and infection, are also significantly higher in AD patients than healthy controls [44]. And clinically, a proteomic study of plasma from control subjects, patients with Mild Cognitive Impairment (MCI) and patients with AD suggested that dysregulated systemic immune responses are present in patients who progress from MCI and go on to develop AD, raising the interesting possibility that this inflammation "communicome" may serve as signature for early detection of the disease [45].

Not only is neuroinflammation believed to be one of the earliest consequences of Aβ deposition, it has been shown to accelerate neurodegeneration and contribute to progression of pathology [46]. Epidemiological studies suggest a link between chronic use of non-steroidal anti-inflammatory drugs (NSAIDs) and reduced risk for AD. In a study of siblings who all eventually developed AD, regular use of NSAIDs delayed the onset of AD and reduced the risk of AD with each year of use [47]. Participants in the Baltimore Longitudinal Aging Study also exhibited a reduced risk for AD with the use of NSAIDs, with those patients who had taken NSAIDs for more than 2 years showing the most reduction in AD risk [48]. Comparison of brains of aged but cognitively normal patients who used NSAIDs chronically with that of cognitively normal patients who did not use NSAIDs revealed no changes in the appearance of senile plaques, but a 3-fold decrease in the number of activated microglia in the brains of chronic NSAID users [49]. Most recently in what is the largest and longest duration epidemiological study to date, long-term (> 5 yrs) NSAID use, in particular ibuprofen, was shown to be protective against development of AD [50]. These findings suggest that the protection afforded by chronic NSAID use in the studies of AD patients may in part be derived by attenuation of microglia activation. Consistent with the epidemiological findings on ibuprofen, chronic ibuprofen administration in aged transgenic mice reduced the number and area of amyloid plaques, as well as the numbers of activated microglia [51], and administration of R-flurbiprofen rescued deficits in hippocampal and medial temporal lobe-dependent memory and learning [52]. However, thus far, clinical trials using systemic administration of NSAIDs have yielded mixed or inconclusive results [53-55], reflecting the need to identify and target the key inflammatory mediators that promote amyloid-associated neuropathology.

However, it is important to note that the presence inflammatory responses may also be necessary to prevent amyloid-associated neurotoxicity. For example, the activation of complement factor C3, the central component of the complement system and a key inflammatory protein may be necessary for plaque clearance by microglia in the AD-afflicted brain [56,57]. In addition, an important component of any inflammatory response is the activation of anti-inflammatory loops that serve to limit and resolve the initial inflammatory response. The Peroxisome Proliferator-Activated Receptor-γ (PPARγ), a nuclear receptor which is activated by metabolites of prostaglandins generated by the COX enzymes and by certain NSAIDs, is upregulated in concert with the COX enzymes in AD brain [42]. As such, PPARγ activation exerts an anti-inflammatory effect, and PPARγ agonists have been shown to inhibit the production of cytokines and pro-inflammatory mediators in response to Aβ [58]. In addition, there appears to be a feedback signaling loop between Aβ and IL-1β: Aβ can induce the production of IL-1β [7], and the presence of IL-1β greatly increases the secretion of cytokines IL-6 and IL-8 in response to Aβ by astrocytes; in the other direction, IL-1α and IL-1β both upregulate the expression of APP, thus probably upregulating the production of Aβ [59]. The ratio of the pro-inflammatory cytokine IL-1β to the anti-inflammatory cytokine IL-10 is drastically elevated in the serum of AD patients, giving these patients a definite pro-inflammatory profile [60]. Increases in levels of IL-1β have been correlated with decreases in LTP in the hippocampus, showing that the cytokine itself may impair memory [61]. Other evidence, however, suggests that IL-1β may not be driving AD pathogenic processes. For example, functional inhibition of IL-1β signaling in mice by genetic ablation of the IL1 receptor [62] or by infusion of IL-1ra did not modulate Aβ deposition. In addition, IL-1β was recently shown to upregulate the α-secretase TACE, thus increasing non-amyloidogenic cleavage of APP and decreasing Aβ production [63]. Therefore, while it may participate in neuroinflammatory responses in AD brain by activating microglia to secrete other inflammatory mediators, IL-1β is not required to drive Aβ deposition and may in fact activate non-amyloidogenic pathways.

An interesting approach to treating amyloid pathology in AD has been the active or passive immunization against Aβ. Immunizing PDAPP mice with Aβ42 prevented or reduced the progression of AD pathology in these mice, depending on whether the immunization took place before or after the development of plaque pathology [64]. Vaccination with Aβ peptides also ameliorated cognitive deficits in mice transgenic for a familial AD (FAD) mutation in APP and an FAD mutation in PS1 [65]. As an alternative delivery route, intranasal administration of Aβ peptides reduced amyloid deposition in AD mouse models [66,67]. Aβ vaccinations were also found successful in reducing Aβ levels in two non-human primates, the rhesus monkey [68] and the Caribbean vervet [69]. A clinical trial of Aβ42 vaccinations was undertaken by Elan Pharmaceuticals in AD patients, and the immunizations resulted in significant clearance of Aβ and plaque removal in some of the patients [70,71], as well as a slowing of the cognitive decline in patients who produced antibodies against the peptide [72]. Unfortunately, the immunization also resulted in dangerous T-lymphocyte meningoencephalitis in some patients, causing the arrest of these trials [70,73]. Passive immunization of PDAPP mice peripherally infused with antibodies that recognize aggregated Aβ in neuritic plaques rapidly increased microglial clustering around plaques detectable by in vivo multi-photon microscopy [74] and reduced plaque burden and AD pathology in PDAPP mice [75]. Administration of antibodies specifically directed against oligomers of Aβ improved learning and memory in Tg2576 mice [76]. Therefore, passive immunization may be a safer approach in AD patients. In addition, there is strong evidence that using shorter Aβ peptides is safer than using full-length Aβ40 or 42 as the immunogen [77]. In short, Aβ immunotherapy holds promise but will need to be made safer and more effective in its ability to generate good antibody titers in the elderly [78]. If these novel immunogens can enhance Aβ antibody generation without the adverse events seen in the earlier clinical trial, neuroimmune modulation by vaccination may become an effective way to prevent AD.

### Parkinson's Disease (PD)

Classically, the neuropathological hallmark of idiopathic PD includes the presence of α-synuclein-positive inclusions in the cell body (Lewy bodies) and processes (Lewy neurites) of specific neurons of the brainstem and a classic motor phenotype resulting from substantial loss of dopaminergic neurons from the substantia nigra pars compacta (SNpc) (reviewed in [79]). A number of studies have confirmed the presence of inflammatory mediators (including TNF, IL-1β, IL-6, and IFNγ) in the cerebrospinal fluid (CSF) of patients with PD as well as in the post-mortem SNpc in PD patient brains [80-84]. Significantly elevated levels of TNF mRNA and protein can be detected in the rodent midbrain substantia nigra within hours of *in vivo *administration of two neurotoxins widely used to model parkinsonism in rodents, 6-hydroxydopamine (6-OHDA) [8] and 1-methyl-4-phenyl-1,2,3,6-tetrahydropyridine (MPTP) [85-87]. Consistent with a role of TNF in contributing to dopaminergic neuron death in chronic parkinsonism, plasma TNF levels were shown to remain elevated in MPTP-treated non-human primates one year after administration of the neurotoxin [88]. In contrast, studies involving mice deficient in TNF or both TNF receptors have yielded conflicting results in that one group reported lack of TNF receptors altered dopamine metabolism and reduced survival of dopaminergic terminals [86] and other groups reported TNF-deficient mice to have reduced sensitivity to MPTP-induced neurotoxicity [85,87]. Loss of TNF receptors versus TNF ligand during development might alter the behavior of microglia or other immune cell populations and contribute to conflicting outcomes in these studies. In addition, the use of different MPTP lesioning regimens in these two studies make it difficult to compare them directly.

Additional evidence that inflammation (and in particular TNF) is involved in nigral DA neuron degeneration comes from two endotoxin rat models. In the first model chronic low dose lipopolysaccharide (LPS) infusion into SNpc of rats results in delayed, selective and progressive loss of nigral DA neurons [89]. In the second model exposure of pregnant rats to LPS and thus, *in utero *exposure of embryos to the endotoxin, caused a loss of DA neurons in postnatal brains [90]. Most importantly, chronic infusion of dominant negative TNF inhibitor proteins into SNpc of adult rats protected nigral DA neurons from LPS and 6-OHDA induced degeneration [91] as did a single nigral injection of a lentivirus encoding DN-TNF in 6-OHDA hemiparkinsonian rats [92]. Given that TNF receptors are expressed in nigrostriatal dopamine neurons [93,94] and these neurons are selectively vulnerable to TNF-induced toxicity [95-99], these early genetic studies and the more recent chronic inflammation models of PD strongly implicate TNF and its downstream targets in neurotoxin- and endotoxin-induced loss of nigral DA neurons. However, because it is clear that the permeability of the BBB increases with age increases the likelihood of peripheral immune cell infiltration into the CNS, TNF produced by brain-resident microglia may not be acting alone in mediating DA neuron cell death but in concert with other circulating neurotoxic factors to increase the inflammatory susceptibility of nigral DA neurons and development of PD.

Another link between inflammation and neurodegeneration comes from studies of single nucleotide polymorphisms that are associated with over-production of cytokines, chemokines and acute phase proteins. These polymorphisms are over-represented in specific cohorts of individuals affected with PD and may confer increased susceptibility for the disease [100-104]. However, most of these findings have not been replicated in independent studies and a meta-analysis of multiple association studies is needed to assess the overall genetic effect of cytokine gene polymorphisms on neurodegenerative disease. Lastly, the advent of technologies such as positron emission tomography (PET) brain scans has enabled clinicians to image microglial activation in living patients. Recent PET studies confirm that patients with idiopathic PD have markedly elevated microglia activation in the pons, basal ganglia, striatum, and frontal and temporal cortical regions irrespective of the number of years with the disease compared to healthy age-matched controls [81]. Persistent activation of the abundant number of microglia in the midbrain region are likely the direct result of elevated levels of cytokines acting in an autocrine manner to potentiate inflammatory responses (e.g., auto-amplification of reactive oxygen species, nitric oxide, and superoxide radicals to form highly oxidizing peroxynitrite species)[7,105-108]. Given that DA neurons in the midbrain have an inherently elevated oxidative intracellular environment as a result of oxidation reactions required for the synthesis of the neurotransmitter dopamine, chronic neuroinflammation is likely to further enhance oxidative stress, hasten dysfunction, and eventually lead to death of DA neurons.

### Huntington's Disease (HD)

Huntington's disease (HD) is an autosomal dominant neurodegenerative disorder that has been linked to mutations in the *huntingtin *gene (htt) [109]. CAG repeat expansions in the htt gene result in an increased number of glutamine residues in the huntingtin protein (polyglutamine expansion). Mutant htt causes degeneration of neurons, predominately in the caudate putamen and cortex, causing HD patients to suffer from uncontrolled movements, emotional disturbances and dementia. While the mechanism by which mutant htt causes neurodegeneration remains obscure, evidence supporting inflammation as an important player in HD is accumulating.

Recent studies have demonstrated that inflammation appears both peripherally and in the central nervous system (CNS) during the progression of HD and HD-like pathology. The R6/2 mouse model of HD displays increased serum levels of IL-6 and in downstream IL-6 effectors, such as alpha-2-macroglobulin (A2 M) and complement components [110]. In the CNS, microarray profiling of several brain regions from HD patients and controls revealed increased gliosis and expression of inflammation-related genes, including GFAP and complement proteins. Increases were most pronounced in the caudate putamen where brain pathology is most severe in HD patients [111]. Increases in labeling of complement proteins in neurons and astrocytes and a 2-5 fold increase in activators and regulators of the classical complement pathway have also been detected in human HD brains by RT-PCR [112]. Lastly, clinical plasma samples from HD gene carriers contain increased levels of pro-inflammatory cytokines involved in the innate immune response, such as IL-6 [113].

A number of studies indicate that the altered immune profile in HD occurs before onset of clinical HD symptoms, suggesting that striatal and cortical neurodegeneration could be exacerbated by inflammation. For example, plasma samples from HD gene carriers contain increased levels of pro-inflammatory cytokines involved in the innate immune response before the onset of clinical symptoms and investigators have been able to discriminate controls from presymptomatic HD mutation carriers by measuring the levels of 3 cytokines, IL-5, IL-6, and IL-10 in plasma [113]. In brains of mutant htt carriers, microglia are activated before onset of symptoms and increased microglial activation correlates with an increased chance of developing HD symptoms in 5 years [114]. Once symptoms have manifested, microglial activation correlates with disease severity [115,116]. In the 3-nitroproprionic acid neurotoxin model of HD in rats, treatment with Celastrol, an anti-inflammatory and antioxidant compound derived from plants, reduced striatal lesion volume [117], indicating that controlling an inflammatory response could be therapeutically beneficial as HD progresses. However, treatment with acetylsalicylate or rofecoxib, anti-inflammatory drugs commonly used in the clinic, was not neuroprotective in either the R6/2 or N171-82Q transgenic mouse models of HD [118].

Since mutant htt is known to cause a number of deleterious effects in cells, it is possible that increased inflammation in HD brains is simply the CNS response to neuronal death that is a direct result of mutant htt toxicity. However, several studies point to the intriguing possibility that mutant htt might itself trigger an inflammatory response, the by-products of which could cause degeneration of neurons. For example, inflammation in HD could be a result of overactive immune cells, such as macrophages in the periphery and microglia in the CNS. Monocytes from HD patients secrete abnormally high levels of the pro-inflammatory cytokine IL-6 in response to a combination of interferon-gamma (IFN-γ) and LPS. Microglia from YAC128 and R6/2 mouse models of HD respond in a similar overactive manner [113]. Mutant htt is found in glial nuclei where it can act to alter gene expression, probably because of its aberrant interactions with transcription factors that are made possible by glutamine expansion-related conformational changes in the htt protein [119]. Interestingly, mutant, but not normal htt activates the IKK complex, the major kinase that leads to phosphorylation-induced degradation of IκBs, leading to increased translocation of NF-κB dimers to the nucleus in mouse striatal cells [120]. Since NF-κB can promote expression of IL-6 and other inflammatory cytokines in glia, such an interaction provides a candidate mechanism by which mutant htt could alter the activity of immune cells leading to an abnormally robust inflammatory response.

The presence of increased inflammatory mediators in the periphery and in the CNS beg the question of whether CNS inflammation "leaks" into the periphery to alter the peripheral immune profile or whether inflammation is triggered in the periphery and immune modulators are allowed to cross the BBB to trigger or contribute to a central inflammatory response. Another possibility is that an inflammatory response is triggered in the periphery and the CNS by an analogous mechanism. These alternatives are just beginning to be explored.

### Amyotrophic Lateral Sclerosis (ALS)

Amyotrophic lateral sclerosis (ALS) involves a progressive degeneration of motor neurons in the brain and spinal cord. While most cases are sporadic in origin, approximately 5-10% of the cases are caused by an autosomal dominant mutation. ALS is typically fatal within 5 years of diagnosis due to a progressive, generalized paralysis that eventually affects the muscles of respiration, causing respiratory failure. Areas where degenerating motor neurons are present in both ALS patients and mouse models are marked by the presence of cytokines and immune cells, including T cells, activated microglia, and astrocytes [121,122]. In addition PET imaging of ALS patients showed an increase in activated microglia in the motor cortex that correlates with upper motor neuron symptoms [123]. In some ALS disease models the presence of immune cells precedes the disease phenotype [124], and the chemokine MCP-1, a potent chemotactic stimulus for microglia [125] is elevated in the CSF of ALS patients [126], suggesting that neuroinflammation could contribute to disease progression. In addition, the levels of general markers of inflammation in the serum of ALS patients correlate positively with the severity of their disability [127]. While multiple genetic loci have been identified as causal in familial forms of ALS, 20% of familial cases involve a gain of function mutation in Cu/Zn superoxide dismutase I (SOD1) [128]. In addition to the well-established role of SOD1 as a critical anti-oxidant enzyme, evidence suggests that part of its normal function is to protect against protein aggregation, a phenomenon that is known to hasten neuronal degeneration. Nevertheless, several studies have suggested that SOD1 mutations in neurons alone are insufficient to cause ALS and that dysfunction in support glia may contribute to disease development and progression [129-132]. For example, ablating microglial expression of mutant SOD 1 (mSOD1) or transplanting bone marrow from wild-type mice into mSOD1 transgenic mice increases the life span of mutant mice [133,134], suggesting that SOD1 mutations may indirectly contribute to neuronal death by affecting glial rather than neuronal function. Consistent with this idea, mSOD1 mice stimulated with LPS secrete more inflammatory mediators, including TNF [135], MCP-1, TGF-β[121] and IFN-γ [136], than control mice. Importantly, levels of TNF correlate with severity of motor neuron loss in mouse models of the disease [137,138] and both TNF receptors are elevated in the serum of ALS patients [139].

In pre-clinical mouse models, certain anti-inflammatory treatments aimed at suppressing microglia activation have been shown to increase the life expectancy of mice expressing human mutant SOD1 by more than 30% [140]. Anti-inflammatory treatments also prevented neurotoxicity when the CSF of ALS patients was applied to rat spinal cord motor neurons [141]. Although a generalized neuroinflammatory response may be driving progressive loss of motor neurons, not all inflammatory mediators have been strongly implicated in ALS. For instance, IL-1β may not be critical to ALS pathogenesis as genetic deletion of IL-1β does not change the lifespan or rate of motor neurodegeneration in mSOD-1 mice [142].

## Summary and Conclusion

It is becoming increasingly evident that neuroinflammation plays a crucial role in the development and progression of many neurodegenerative diseases. Glia and in particular microglia are central to mediating the effects of neuroinflammation. While neuroinflammation and microglia provide an attractive therapeutic target in the treatment and prevention of neurodegenerative diseases investigators face several challenges ahead (Appendix 2) which must be overcome before one can advocate in favor of large-scale anti-inflammatory trials in the clinic. Some of these include developing approaches to improve the access of drugs to CNS tissue as well as developing therapies that maintain or optimize the beneficial effects of neuroinflammation while eliminating or minimizing its detrimental effects.

## Competing interests

The authors declare that they have no competing interests.

## Authors' contributions

MGT: primary manuscript content, primary writing of Parkinson's disease, shared primary writing of overview, and secondary contributions to microglial activation and Alzheimer's disease. TCFC: primary writing of microglia activation, ALS, and conclusions, and shared primary writing of overview. LTA: primary writing of Huntington's disease, secondary contributions to Parkinson's and Alzheimer's disease sections. FEM: primary writing of Alzheimer's disease and secondary contributions to ALS section. All authors' contributed to editing and revision of manuscript and have read and approved the final manuscript.

## Appendix 1: Key Observations

1. Neurodegenerative diseases are associated with signs of chronic neuroinflammation

2. A variety of initiating triggers (some as yet unknown) associated with the different neurodegenerative disorders converge at a common intersection point - activation of microglia.

3. While the initial neuroimmune response may be aimed at limiting the disease process, chronic neuroinflammation driven by persistent microglia activation is likely to aid in the progression of the disease and the hastening of neuronal demise.

4. How the inflammatory response affects specific neuronal and glial populations and contributes to specific neurodegenerative diseases remains a critical and unanswered question.

## Appendix 2: Critical challenges involved in developing neuroprotective anti-inflammatory therapeutic strategies

1. Identify internal and external factors that trigger chronic neuroinflammatory responses, with a focus on how acute immune responses become chronic.

2. Identify inflammatory mediators that compromise survival of specific neuronal populations.

3. Develop therapeutic compounds that cross the blood brain barrier (BBB)

4. Selectively target destructive inflammatory mediators without compromising beneficial survival-promoting effects and overall immune function.

5. Develop inclusion and exclusion criteria for human subjects to be enrolled in clinical trials taking into account their immune status.

## References

[B1] Rivest S Regulation of innate immune responses in the brain Nat Rev Immunol 2009 9 6 429 39 19461673 10.1038/nri2565

[B2] Crutcher KA Gendelman HE Kipnis J Perez-Polo JR Perry VH Popovich PG Weaver LC Debate: "is increasing neuroinflammation beneficial for neural repair?" J Neuroimmune Pharmacol 2006 1 3 195 211 18040798 10.1007/s11481-006-9021-7

[B3] Popovich PG Longbrake EE Can the immune system be harnessed to repair the CNS? Nat Rev Neurosci 2008 9 6 481 93 18490917 10.1038/nrn2398

[B4] Tansey MG McCoy MK Frank-Cannon TC Neuroinflammatory mechanisms in Parkinson's disease: potential environmental triggers, pathways, and targets for early therapeutic intervention Exp Neurol 2007 208 1 1 25 17720159 10.1016/j.expneurol.2007.07.004 PMC3707134

[B5] Block ML Hong JS Microglia and inflammation-mediated neurodegeneration: multiple triggers with a common mechanism Prog Neurobiol 2005 76 2 77 98 16081203 10.1016/j.pneurobio.2005.06.004

[B6] McGeer EG McGeer PL The role of anti-inflammatory agents in Parkinson's disease CNS Drugs 2007 21 10 789 97 17850169 10.2165/00023210-200721100-00001

[B7] Mrak RE Griffin WS Glia and their cytokines in progression of neurodegeneration Neurobiol Aging 2005 26 3 349 54 15639313 10.1016/j.neurobiolaging.2004.05.010

[B8] Nagatsu T Sawada M Cellular and molecular mechanisms of Parkinson's disease: neurotoxins, causative genes, and inflammatory cytokines Cell Mol Neurobiol 2006 26 4-6 781 802 16823625 10.1007/s10571-006-9061-9 PMC11520651

[B9] Tansey MG Wyss-Coray T Lane M, Bergmann C, Wyss-Coray T Cytokines in CNS Inflammation and Disease Central Nervous System Diseases and Inflammation, T.E.C 2008 Springer: New York 59 106

[B10] Schmid CD Melchior B Masek K Puntambekar SS Danielson PE Lo DD Sutcliffe JG Carson MJ Differential gene expression in LPS/IFNgamma activated microglia and macrophages: in vitro versus in vivo J Neurochem 2009 109 Suppl 1 117 25 19393017 10.1111/j.1471-4159.2009.05984.x PMC2766614

[B11] Nee LE Polinsky RJ Eldridge R Weingartner H Smallberg S Ebert M A family with histologically confirmed Alzheimer's disease Arch Neurol 1983 40 4 203 8 6600923 10.1001/archneur.1983.04050040033004

[B12] Goate A Chartier-Harlin MC Mullan M Brown J Crawford F Fidani L Giuffra L Haynes A Irving N James L Mant R Newton P Rooke K Roques P Talbot C Pericak-Vance M Roses A Williamson R Rossor M Owen M Hardy J Segregation of a missense mutation in the amyloid precursor protein gene with familial Alzheimer's disease Nature 1991 349 6311 704 6 1671712 10.1038/349704a0

[B13] Citron M Oltersdorf T Haass C McConlogue L Hung AY Seubert P Vigo-Pelfrey C Lieberburg I Selkoe DJ Mutation of the beta-amyloid precursor protein in familial Alzheimer's disease increases beta-protein production Nature 1992 360 6405 672 4 1465129 10.1038/360672a0

[B14] Cai XD Golde TE Younkin SG Release of excess amyloid beta protein from a mutant amyloid beta protein precursor Science 1993 259 5094 514 6 8424174 10.1126/science.8424174

[B15] Lopera F Ardilla A Martinez A Madrigal L Arango-Viana JC Lemere CA Arango-Lasprilla JC Hincapie L Arcos-Burgos M Ossa JE Behrens IM Norton J Lendon C Goate AM Ruiz-Linares A Rosselli M Kosik KS Clinical features of early-onset Alzheimer disease in a large kindred with an E280A presenilin-1 mutation JAMA 1997 277 10 793 9 9052708

[B16] Lemere CA Lopera F Kosik KS Lendon CL Ossa J Saido TC Yamaguchi H Ruiz A Martinez A Madrigal L Hincapie L Arango JC Anthony DC Koo EH Goate AM Selkoe DJ The E280A presenilin 1 Alzheimer mutation produces increased A beta 42 deposition and severe cerebellar pathology Nat Med 1996 2 10 1146 50 8837617 10.1038/nm1096-1146

[B17] Whalen BM Selkoe DJ Hartley DM Small non-fibrillar assemblies of amyloid beta-protein bearing the Arctic mutation induce rapid neuritic degeneration Neurobiol Dis 2005 20 2 254 66 16242634 10.1016/j.nbd.2005.03.007

[B18] McGeer PL Itagaki S Tago H McGeer EG Reactive microglia in patients with senile dementia of the Alzheimer type are positive for the histocompatibility glycoprotein HLA-DR Neurosci Lett 1987 79 1-2 195 200 3670729 10.1016/0304-3940(87)90696-3

[B19] Akiyama H Barger S Barnum S Bradt B Bauer J Cole GM Cooper NR Eikelenboom P Emmerling M Fiebich BL Finch CE Frautschy S Griffin WS Hampel H Hull M Landreth G Lue L Mrak R Mackenzie IR McGeer PL O'Banion MK Pachter J Pasinetti G Plata-Salaman C Rogers J Rydel R Shen Y Streit W Strohmeyer R Tooyoma I Van Muiswinkel FL Veerhuis R Walker D Webster S Wegrzyniak B Wenk G Wyss-Coray T Inflammation and Alzheimer's disease Neurobiol Aging 2000 21 3 383 421 10858586 10.1016/s0197-4580(00)00124-x PMC3887148

[B20] Eikelenboom P Veerhuis R Scheper W Rozemuller AJ van Gool WA Hoozemans JJ The significance of neuroinflammation in understanding Alzheimer's disease J Neural Transm 2006 113 11 1685 95 17036175 10.1007/s00702-006-0575-6

[B21] Griffin WS Inflammation and neurodegenerative diseases Am J Clin Nutr 2006 83 2 470S 474S 16470015 10.1093/ajcn/83.2.470S

[B22] Hoozemans JJ Veerhuis R Rozemuller JM Eikelenboom P Neuroinflammation and regeneration in the early stages of Alzheimer's disease pathology Int J Dev Neurosci 2006 24 2-3 157 65 16384684 10.1016/j.ijdevneu.2005.11.001

[B23] McGeer EG McGeer PL Inflammatory processes in Alzheimer's disease Prog Neuropsychopharmacol Biol Psychiatry 2003 27 5 741 9 12921904 10.1016/S0278-5846(03)00124-6

[B24] Wyss-Coray T Mucke L Inflammation in neurodegenerative disease--a double-edged sword Neuron 2002 35 3 419 32 12165466 10.1016/s0896-6273(02)00794-8

[B25] McAlpine FE Lee JK Harms AS Ruhn KA Blurton-Jones M Hong J Das P Golde TE LaFerla FM Oddo S Blesch A Tansey MG Inhibition of soluble TNF signaling in a mouse model of Alzheimer's disease prevents pre-plaque amyloid-associated neuropathology Neurobiol Dis 2009 34 1 163 77 19320056 10.1016/j.nbd.2009.01.006 PMC2948857

[B26] Eikelenboom P Bate C Van Gool WA Hoozemans JJ Rozemuller JM Veerhuis R Williams A Neuroinflammation in Alzheimer's disease and prion disease Glia 2002 40 2 232 9 12379910 10.1002/glia.10146

[B27] Bate C Veerhuis R Eikelenboom P Williams A Microglia kill amyloid-beta1-42 damaged neurons by a CD14-dependent process Neuroreport 2004 15 9 1427 30 15194867 10.1097/01.wnr.0000132203.76836.16

[B28] Ard MD Cole GM Wei J Mehrle AP Fratkin JD Scavenging of Alzheimer's amyloid beta-protein by microglia in culture J Neurosci Res 1996 43 2 190 202 8820967 10.1002/(SICI)1097-4547(19960115)43:2<190::AID-JNR7>3.0.CO;2-B

[B29] Kopec KK Carroll RT Alzheimer's beta-amyloid peptide 1-42 induces a phagocytic response in murine microglia J Neurochem 1998 71 5 2123 31 9798938 10.1046/j.1471-4159.1998.71052123.x

[B30] Shaffer LM Dority MD Gupta-Bansal R Frederickson RC Younkin SG Brunden KR Amyloid beta protein (A beta) removal by neuroglial cells in culture Neurobiol Aging 1995 16 5 737 45 8532106 10.1016/0197-4580(95)00055-j

[B31] Koenigsknecht-Talboo J Landreth GE Microglial phagocytosis induced by fibrillar beta-amyloid and IgGs are differentially regulated by proinflammatory cytokines J Neurosci 2005 25 36 8240 9 16148231 10.1523/JNEUROSCI.1808-05.2005 PMC6725530

[B32] Sheng JG Griffin WS Royston MC Mrak RE Distribution of interleukin-1-immunoreactive microglia in cerebral cortical layers: implications for neuritic plaque formation in Alzheimer's disease Neuropathol Appl Neurobiol 1998 24 4 278 83 9775393 10.1046/j.1365-2990.1998.00122.x PMC3833591

[B33] Frautschy SA Yang F Irrizarry M Hyman B Saido TC Hsiao K Cole GM Microglial response to amyloid plaques in APPsw transgenic mice Am J Pathol 1998 152 1 307 17 9422548 PMC1858113

[B34] Mackenzie IR Hao C Munoz DG Role of microglia in senile plaque formation Neurobiol Aging 1995 16 5 797 804 8532113 10.1016/0197-4580(95)00092-s

[B35] Benzing WC Wujek JR Ward EK Shaffer D Ashe KH Younkin SG Brunden KR Evidence for glial-mediated inflammation in aged APP(SW) transgenic mice Neurobiol Aging 1999 20 6 581 9 10674423 10.1016/s0197-4580(99)00065-2

[B36] Flick DA Gifford GE Production of tumor necrosis factor in unprimed mice: mechanism of endotoxin-mediated tumor necrosis Immunobiology 1986 171 4-5 320 8 3527951 10.1016/S0171-2985(86)80064-X

[B37] Qiao X Cummins DJ Paul SM Neuroinflammation-induced acceleration of amyloid deposition in the APPV717F transgenic mouse Eur J Neurosci 2001 14 3 474 82 11553297 10.1046/j.0953-816x.2001.01666.x

[B38] Sheng JG Bora SH Xu G Borchelt DR Price DL Koliatsos VE Lipopolysaccharide-induced-neuroinflammation increases intracellular accumulation of amyloid precursor protein and amyloid beta peptide in APPswe transgenic mice Neurobiol Dis 2003 14 1 133 45 13678674 10.1016/s0969-9961(03)00069-x

[B39] Kitazawa M Oddo S Yamasaki TR Green KN LaFerla FM Lipopolysaccharide-induced inflammation exacerbates tau pathology by a cyclin-dependent kinase 5-mediated pathway in a transgenic model of Alzheimer's disease J Neurosci 2005 25 39 8843 53 16192374 10.1523/JNEUROSCI.2868-05.2005 PMC6725603

[B40] Wyss-Coray T Masliah E Mallory M McConlogue L Johnson-Wood K Lin C Mucke L Amyloidogenic role of cytokine TGF-beta1 in transgenic mice and in Alzheimer's disease Nature 1997 389 6651 603 6 9335500 10.1038/39321

[B41] Harris-White ME Chu T Balverde Z Sigel JJ Flanders KC Frautschy SA Effects of transforming growth factor-beta (isoforms 1-3) on amyloid-beta deposition, inflammation, and cell targeting in organotypic hippocampal slice cultures J Neurosci 1998 18 24 10366 74 9852574 10.1523/JNEUROSCI.18-24-10366.1998 PMC6793343

[B42] Kitamura Y Shimohama S Koike H Kakimura J Matsuoka Y Nomura Y Gebicke-Haerter PJ Taniguchi T Increased expression of cyclooxygenases and peroxisome proliferator-activated receptor-gamma in Alzheimer's disease brains Biochem Biophys Res Commun 1999 254 3 582 6 9920782 10.1006/bbrc.1998.9981

[B43] Lukiw WJ Bazan NG Neuroinflammatory signaling upregulation in Alzheimer's disease Neurochem Res 2000 25 9-10 1173 84 11059791 10.1023/a:1007627725251

[B44] Lieberman J Schleissner L Tachiki KH Kling AS Serum alpha 1-antichymotrypsin level as a marker for Alzheimer-type dementia Neurobiol Aging 1995 16 5 747 53 8532107 10.1016/0197-4580(95)00056-k

[B45] Ray S Britschgi M Herbert C Takeda-Uchimura Y Boxer A Blennow K Friedman LF Galasko DR Jutel M Karydas A Kaye JA Leszek J Miller BL Minthon L Quinn JF Rabinovici GD Robinson WH Sabbagh MN So YT Sparks DL Tabaton M Tinklenberg J Yesavage JA Tibshirani R Wyss-Coray T Classification and prediction of clinical Alzheimer's diagnosis based on plasma signaling proteins Nat Med 2007 13 11 1359 62 17934472 10.1038/nm1653

[B46] Craft JM Watterson DM Van Eldik LJ Human amyloid beta-induced neuroinflammation is an early event in neurodegeneration Glia 2006 53 5 484 90 16369931 10.1002/glia.20306

[B47] Breitner JC Welsh KA Helms MJ Gaskell PC Gau BA Roses AD Pericak-Vance MA Saunders AM Delayed onset of Alzheimer's disease with nonsteroidal anti-inflammatory and histamine H2 blocking drugs Neurobiol Aging 1995 16 4 523 30 8544901 10.1016/0197-4580(95)00049-k

[B48] Stewart WF Kawas C Corrada M Metter EJ Risk of Alzheimer's disease and duration of NSAID use Neurology 1997 48 3 626 32 9065537 10.1212/wnl.48.3.626

[B49] Mackenzie IR Munoz DG Nonsteroidal anti-inflammatory drug use and Alzheimer-type pathology in aging Neurology 1998 50 4 986 90 9566383 10.1212/wnl.50.4.986

[B50] Vlad SC Miller DR Kowall NW Felson DT Protective effects of NSAIDs on the development of Alzheimer disease Neurology 2008 70 19 1672 7 18458226 10.1212/01.wnl.0000311269.57716.63 PMC2758242

[B51] Lim GP Yang F Chu T Chen P Beech W Teter B Tran T Ubeda O Ashe KH Frautschy SA Cole GM Ibuprofen suppresses plaque pathology and inflammation in a mouse model for Alzheimer's disease J Neurosci 2000 20 15 5709 14 10908610 10.1523/JNEUROSCI.20-15-05709.2000 PMC6772529

[B52] Kukar T Prescott S Eriksen JL Holloway V Murphy MP Koo EH Golde TE Nicolle MM Chronic administration of R-flurbiprofen attenuates learning impairments in transgenic amyloid precursor protein mice BMC Neurosci 2007 8 54 17650315 10.1186/1471-2202-8-54 PMC1948891

[B53] Launer L Nonsteroidal anti-inflammatory drug use and the risk for Alzheimer's disease: dissecting the epidemiological evidence Drugs 2003 63 8 731 9 12662122 10.2165/00003495-200363080-00001

[B54] McGeer PL McGeer EG NSAIDs and Alzheimer disease: epidemiological, animal model and clinical studies Neurobiol Aging 2007 28 5 639 47 16697488 10.1016/j.neurobiolaging.2006.03.013

[B55] van Gool WA Aisen PS Eikelenboom P Anti-inflammatory therapy in Alzheimer's disease: is hope still alive? J Neurol 2003 250 7 788 92 12883918 10.1007/s00415-003-1146-5

[B56] Maier M Peng Y Jiang L Seabrook TJ Carroll MC Lemere CA Complement C3 deficiency leads to accelerated amyloid beta plaque deposition and neurodegeneration and modulation of the microglia/macrophage phenotype in amyloid precursor protein transgenic mice J Neurosci 2008 28 25 6333 41 18562603 10.1523/JNEUROSCI.0829-08.2008 PMC3329761

[B57] Wyss-Coray T Yan F Lin AH Lambris JD Alexander JJ Quigg RJ Masliah E Prominent neurodegeneration and increased plaque formation in complement-inhibited Alzheimer's mice Proc Natl Acad Sci USA 2002 99 16 10837 42 12119423 10.1073/pnas.162350199 PMC125059

[B58] Combs CK Johnson DE Karlo JC Cannady SB Landreth GE Inflammatory mechanisms in Alzheimer's disease: inhibition of beta-amyloid-stimulated proinflammatory responses and neurotoxicity by PPARgamma agonists J Neurosci 2000 20 2 558 67 10632585 10.1523/JNEUROSCI.20-02-00558.2000 PMC6772401

[B59] Sheng JG Ito K Skinner RD Mrak RE Rovnaghi CR Van Eldik LJ Griffin WS In vivo and in vitro evidence supporting a role for the inflammatory cytokine interleukin-1 as a driving force in Alzheimer pathogenesis Neurobiol Aging 1996 17 5 761 6 8892349 10.1016/0197-4580(96)00104-2 PMC3886636

[B60] Remarque EJ Bollen EL Weverling-Rijnsburger AW Laterveer JC Blauw GJ Westendorp RG Patients with Alzheimer's disease display a pro-inflammatory phenotype Exp Gerontol 2001 36 1 171 6 11162920 10.1016/s0531-5565(00)00176-5

[B61] Lynch MA Age-related impairment in long-term potentiation in hippocampus: a role for the cytokine, interleukin-1 beta? Prog Neurobiol 1998 56 5 571 89 9775404 10.1016/s0301-0082(98)00054-9

[B62] Das P Smithson LA Price RW Holloway VM Levites Y Chakrabarty P Golde TE Interleukin-1 receptor 1 knockout has no effect on amyloid deposition in Tg2576 mice and does not alter efficacy following Abeta immunotherapy J Neuroinflammation 2006 3 17 16872492 10.1186/1742-2094-3-17 PMC1559596

[B63] Tachida Y Nakagawa K Saito T Saido TC Honda T Saito Y Murayama S Endo T Sakaguchi G Kato A Kitazume S Hashimoto Y Interleukin-1 beta up-regulates TACE to enhance alpha-cleavage of APP in neurons: resulting decrease in Abeta production J Neurochem 2008 104 5 1387 93 18021299 10.1111/j.1471-4159.2007.05127.x

[B64] Schenk D Barbour R Dunn W Gordon G Grajeda H Guido T Hu K Huang J Johnson-Wood K Khan K Kholodenko D Lee M Liao Z Lieberburg I Motter R Mutter L Soriano F Shopp G Vasquez N Vandevert C Walker S Wogulis M Yednock T Games D Seubert P Immunization with amyloid-beta attenuates Alzheimer-disease-like pathology in the PDAPP mouse Nature 1999 400 6740 173 7 10408445 10.1038/22124

[B65] Morgan D Diamond DM Gottschall PE Ugen KE Dickey C Hardy J Duff K Jantzen P DiCarlo G Wilcock D Connor K Hatcher J Hope C Gordon M Arendash GW A beta peptide vaccination prevents memory loss in an animal model of Alzheimer's disease Nature 2000 408 6815 982 5 11140686 10.1038/35050116

[B66] Lemere CA Maron R Selkoe DJ Weiner HL Nasal vaccination with beta-amyloid peptide for the treatment of Alzheimer's disease DNA Cell Biol 2001 20 11 705 11 11788048 10.1089/10445490152717569

[B67] Lemere CA Spooner ET Leverone JF Mori C Clements JD Intranasal immunotherapy for the treatment of Alzheimer's disease: Escherichia coli LT and LT(R192G) as mucosal adjuvants Neurobiol Aging 2002 23 6 991 1000 12470794 10.1016/s0197-4580(02)00127-6

[B68] Gandy S DeMattos RB Lemere CA Heppner FL Leverone J Aguzzi A Ershler WB Dai J Fraser P St George Hyslop P Holtzman DM Walker LC Keller ET Alzheimer's Abeta vaccination of rhesus monkeys (Macaca mulatta) Mech Ageing Dev 2004 125 2 149 51 15037022 10.1016/j.mad.2003.12.002

[B69] Lemere CA Beierschmitt A Iglesias M Spooner ET Bloom JK Leverone JF Zheng JB Seabrook TJ Louard D Li D Selkoe DJ Palmour RM Ervin FR Alzheimer's disease abeta vaccine reduces central nervous system abeta levels in a non-human primate, the Caribbean vervet Am J Pathol 2004 165 1 283 97 15215183 10.1016/s0002-9440(10)63296-8 PMC1618542

[B70] Nicoll JA Barton E Boche D Neal JW Ferrer I Thompson P Vlachouli C Wilkinson D Bayer A Games D Seubert P Schenk D Holmes C Abeta species removal after abeta42 immunization J Neuropathol Exp Neurol 2006 65 11 1040 8 17086100 10.1097/01.jnen.0000240466.10758.ce

[B71] Nicoll JA Wilkinson D Holmes C Steart P Markham H Weller RO Neuropathology of human Alzheimer disease after immunization with amyloid-beta peptide: a case report Nat Med 2003 9 4 448 52 12640446 10.1038/nm840

[B72] Hock C Konietzko U Streffer JR Tracy J Signorell A Muller-Tillmanns B Lemke U Henke K Moritz E Garcia E Wollmer MA Umbricht D de Quervain DJ Hofmann M Maddalena A Papassotiropoulos A Nitsch RM Antibodies against beta-amyloid slow cognitive decline in Alzheimer's disease Neuron 2003 38 4 547 54 12765607 10.1016/s0896-6273(03)00294-0

[B73] Orgogozo JM Gilman S Dartigues JF Laurent B Puel M Kirby LC Jouanny P Dubois B Eisner L Flitman S Michel BF Boada M Frank A Hock C Subacute meningoencephalitis in a subset of patients with AD after Abeta42 immunization Neurology 2003 61 1 46 54 12847155 10.1212/01.wnl.0000073623.84147.a8

[B74] Koenigsknecht-Talboo J Meyer-Luehmann M Parsadanian M Garcia-Alloza M Finn MB Hyman BT Bacskai BJ Holtzman DM Rapid microglial response around amyloid pathology after systemic anti-Abeta antibody administration in PDAPP mice J Neurosci 2008 28 52 14156 64 19109498 10.1523/JNEUROSCI.4147-08.2008 PMC2743894

[B75] Bard F Cannon C Barbour R Burke RL Games D Grajeda H Guido T Hu K Huang J Johnson-Wood K Khan K Kholodenko D Lee M Lieberburg I Motter R Nguyen M Soriano F Vasquez N Weiss K Welch B Seubert P Schenk D Yednock T Peripherally administered antibodies against amyloid beta-peptide enter the central nervous system and reduce pathology in a mouse model of Alzheimer disease Nat Med 2000 6 8 916 9 10932230 10.1038/78682

[B76] Lee EB Leng LZ Zhang B Kwong L Trojanowski JQ Abel T Lee VM Targeting amyloid-beta peptide (Abeta) oligomers by passive immunization with a conformation-selective monoclonal antibody improves learning and memory in Abeta precursor protein (APP) transgenic mice J Biol Chem 2006 281 7 4292 9 16361260 10.1074/jbc.M511018200

[B77] Lemere CA Maier M Peng Y Jiang L Seabrook TJ Novel Abeta immunogens: is shorter better? Curr Alzheimer Res 2007 4 4 427 36 17908047 10.2174/156720507781788800

[B78] Lemere CA Developing novel immunogens for a safe and effective Alzheimer's disease vaccine Prog Brain Res 2009 175 83 93 19660650 10.1016/S0079-6123(09)17506-4 PMC2814339

[B79] Litvan I Halliday G Hallett M Goetz CG Rocca W Duyckaerts C Ben-Shlomo Y Dickson DW Lang AE Chesselet MF Langston WJ Di Monte DA Gasser T Hagg T Hardy J Jenner P Melamed E Myers RH Parker D Jr Price DL The etiopathogenesis of Parkinson disease and suggestions for future research. Part I J Neuropathol Exp Neurol 2007 66 4 251 7 17413315 10.1097/nen.0b013e3180415e42

[B80] Banati RB Daniel SE Blunt SB Glial pathology but absence of apoptotic nigral neurons in long-standing Parkinson's disease Mov Disord 1998 13 2 221 7 9539333 10.1002/mds.870130205

[B81] Gerhard A Pavese N Hotton G Turkheimer F Es M Hammers A Eggert K Oertel W Banati RB Brooks DJ In vivo imaging of microglial activation with [11C](R)-PK11195 PET in idiopathic Parkinson's disease Neurobiol Dis 2006 21 2 404 12 16182554 10.1016/j.nbd.2005.08.002

[B82] Hunot S Dugas N Faucheux B Hartmann A Tardieu M Debre P Agid Y Dugas B Hirsch EC FcepsilonRII/CD23 is expressed in Parkinson's disease and induces, in vitro, production of nitric oxide and tumor necrosis factor-alpha in glial cells J Neurosci 1999 19 9 3440 7 10212304 10.1523/JNEUROSCI.19-09-03440.1999 PMC6782235

[B83] McGeer PL Itagaki S Boyes BE McGeer EG Reactive microglia are positive for HLA-DR in the substantia nigra of Parkinson's and Alzheimer's disease brains Neurology 1988 38 8 1285 91 3399080 10.1212/wnl.38.8.1285

[B84] Vawter MP Dillon-Carter O Tourtellotte WW Carvey P Freed WJ TGFbeta1 and TGFbeta2 concentrations are elevated in Parkinson's disease in ventricular cerebrospinal fluid Exp Neurol 1996 142 2 313 22 8934562 10.1006/exnr.1996.0200

[B85] Ferger B Leng A Mura A Hengerer B Feldon J Genetic ablation of tumor necrosis factor-alpha (TNF-alpha) and pharmacological inhibition of TNF-synthesis attenuates MPTP toxicity in mouse striatum J Neurochem 2004 89 4 822 33 15140182 10.1111/j.1471-4159.2004.02399.x

[B86] Rousselet E Callebert J Parain K Joubert C Hunot S Hartmann A Jacque C Perez-Diaz F Cohen-Salmon C Launay JM Hirsch EC Role of TNF-alpha receptors in mice intoxicated with the parkinsonian toxin MPTP Exp Neurol 2002 177 1 183 92 12429221 10.1006/exnr.2002.7960

[B87] Sriram K Matheson JM Benkovic SA Miller DB Luster MI O'Callaghan JP Mice deficient in TNF receptors are protected against dopaminergic neurotoxicity: implications for Parkinson's disease Faseb J 2002 16 11 1474 1476 12205053 10.1096/fj.02-0216fje

[B88] Barcia C de Pablos V Bautista-Hernandez V Sanchez-Bahillo A Bernal I Fernandez-Villalba E Martin J Banon R Fernandez-Barreiro A Herrero MT Increased plasma levels of TNF-alpha but not of IL1-beta in MPTP-treated monkeys one year after the MPTP administration Parkinsonism Relat Disord 2005 11 7 435 439 16154791 10.1016/j.parkreldis.2005.05.006

[B89] Gao HM Jiang J Wilson B Zhang W Hong JS Liu B Microglial activation-mediated delayed and progressive degeneration of rat nigral dopaminergic neurons: relevance to Parkinson's disease J Neurochem 2002 81 6 1285 1297 12068076 10.1046/j.1471-4159.2002.00928.x

[B90] Ling Z Gayle DA Ma SY Lipton JW Tong CW Hong JS Carvey PM In utero bacterial endotoxin exposure causes loss of tyrosine hydroxylase neurons in the postnatal rat midbrain Mov Disord 2002 17 1 116 124 11835448 10.1002/mds.10078

[B91] McCoy MK Martinez TN Ruhn KA Szymkowski DE Smith CG Botterman BR Tansey KE Tansey MG Blocking soluble tumor necrosis factor signaling with dominant-negative tumor necrosis factor inhibitor attenuates loss of dopaminergic neurons in models of Parkinson's disease J Neurosci 2006 26 37 9365 9375 16971520 10.1523/JNEUROSCI.1504-06.2006 PMC3707118

[B92] McCoy MK Ruhn KA Martinez TN McAlpine FE Blesch A Tansey MG Intranigral lentiviral delivery of dominant-negative TNF attenuates neurodegeneration and behavioral deficits in hemiparkinsonian rats Mol Ther 2008 16 9 1572 1579 18628756 10.1038/mt.2008.146 PMC2670754

[B93] Boka G Anglade P Wallach D Javoy-Agid F Agid Y Hirsch EC Immunocytochemical analysis of tumor necrosis factor and its receptors in Parkinson's disease Neurosci Lett 1994 172 1-2 151 154 8084523 10.1016/0304-3940(94)90684-x

[B94] Tartaglia LA Rothe M Hu YF Goeddel DV Tumor necrosis factor's cytotoxic activity is signaled by the p55 TNF receptor Cell 1993 73 2 213 216 8386591 10.1016/0092-8674(93)90222-c

[B95] Aloe L Fiore M TNF-alpha expressed in the brain of transgenic mice lowers central tyroxine hydroxylase immunoreactivity and alters grooming behavior Neurosci Lett 1997 238 1-2 65 68 9464656 10.1016/s0304-3940(97)00850-1

[B96] Carvey PM Chen EY Lipton JW Tong CW Chang QA Ling ZD Intra-parenchymal injection of tumor necrosis factor-alpha and interleukin 1-beta produces dopamine neuron loss in the rat J Neural Transm 2005 112 5 601 612 15583962 10.1007/s00702-004-0222-z

[B97] Gayle DA Ling Z Tong C Landers T Lipton JW Carvey PM Lipopolysaccharide (LPS)-induced dopamine cell loss in culture: roles of tumor necrosis factor-alpha, interleukin-1beta, and nitric oxide Brain Res Dev Brain Res 2002 133 1 27 35 11850061 10.1016/s0165-3806(01)00315-7

[B98] Ling ZD Potter ED Lipton JW Carvey PM Differentiation of mesencephalic progenitor cells into dopaminergic neurons by cytokines Exp Neurol 1998 149 2 411 423 9500954 10.1006/exnr.1998.6715

[B99] McGuire SO Ling ZD Lipton JW Sortwell CE Collier TJ Carvey PM Tumor necrosis factor alpha is toxic to embryonic mesencephalic dopamine neurons Exp Neurol 2001 169 2 219 230 11358437 10.1006/exnr.2001.7688

[B100] Hakansson A Westberg L Nilsson S Buervenich S Carmine A Holmberg B Sydow O Olson L Johnels B Eriksson E Investigation of genes coding for inflammatory components in Parkinson's disease Mov Disord 2005 20 5 569 573 15648059 10.1002/mds.20378

[B101] Hakansson A Westberg L Nilsson S Buervenich S Carmine A Holmberg B Sydow O Olson L Johnels B Eriksson E Interaction of polymorphisms in the genes encoding interleukin-6 and estrogen receptor beta on the susceptibility to Parkinson's disease Am J Med Genet B Neuropsychiatr Genet 2005 133 1 88 92 10.1002/ajmg.b.30136 15635591

[B102] Kruger R Hardt C Tschentscher F Jackel S Kuhn W Muller T Werner J Woitalla D Berg D Kuhnl N Genetic analysis of immunomodulating factors in sporadic Parkinson's disease J Neural Transm 2000 107 5 553 562 11072751 10.1007/s007020070078

[B103] Nishimura M Kuno S Kaji R Yasuno K Kawakami H Glutathione-S-transferase-1 and interleukin-1beta gene polymorphisms in Japanese patients with Parkinson's disease Mov Disord 2005 20 7 901 902 15834859 10.1002/mds.20477

[B104] Nishimura M Mizuta I Mizuta E Yamasaki S Ohta M Kaji R Kuno S Tumor necrosis factor gene polymorphisms in patients with sporadic Parkinson's disease Neurosci Lett 2001 311 1 1 4 11585553 10.1016/s0304-3940(01)02111-5

[B105] Block ML Zecca L Hong JS Microglia-mediated neurotoxicity: uncovering the molecular mechanisms Nat Rev Neurosci 2007 8 1 57 69 17180163 10.1038/nrn2038

[B106] Ito S Sawada M Haneda M Ishida Y Isobe K Amyloid-beta peptides induce several chemokine mRNA expressions in the primary microglia and Ra2 cell line via the PI3K/Akt and/or ERK pathway Neurosci Res 2006 56 3 294 299 16978723 10.1016/j.neures.2006.07.009

[B107] Kim YS Joh TH Microglia, major player in the brain inflammation: their roles in the pathogenesis of Parkinson's disease Exp Mol Med 2006 38 4 333 347 16953112 10.1038/emm.2006.40

[B108] Sawada M Imamura K Nagatsu T Role of cytokines in inflammatory process in Parkinson's disease J Neural Transm Suppl 2006 70 373 381 17017556 10.1007/978-3-211-45295-0_57

[B109] Group THsDCR A novel gene containing a trinucleotide repeat that is expanded and unstable on Huntington's disease chromosomes Cell 1993 72 971 983 8458085 10.1016/0092-8674(93)90585-e

[B110] Dalrymple A Wild EJ Joubert R Sathasivam K Bjorkqvist M Petersen A Jackson GS Isaacs JD Kristiansen M Bates GP Proteomic profiling of plasma in Huntington's disease reveals neuroinflammatory activation and biomarker candidates Journal of proteome research 2007 6 7 2833 2840 17552550 10.1021/pr0700753

[B111] Hodges A Strand AD Aragaki AK Kuhn A Sengstag T Hughes G Elliston LA Hartog C Goldstein DR Thu D Regional and cellular gene expression changes in human Huntington's disease brain Hum Mol Genet 2006 15 6 965 977 16467349 10.1093/hmg/ddl013

[B112] Singhrao SK Neal JW Morgan BP Gasque P Increased complement biosynthesis by microglia and complement activation on neurons in Huntington's disease Exp Neurol 1999 159 2 362 376 10506508 10.1006/exnr.1999.7170

[B113] Bjorkqvist M Wild EJ Thiele J Silvestroni A Andre R Lahiri N Raibon E Lee RV Benn CL Soulet D A novel pathogenic pathway of immune activation detectable before clinical onset in Huntington's disease J Exp Med 2008 205 8 1869 1877 18625748 10.1084/jem.20080178 PMC2525598

[B114] Tai YF Pavese N Gerhard A Tabrizi SJ Barker RA Brooks DJ Piccini P Microglial activation in presymptomatic Huntington's disease gene carriers Brain 2007 130 Pt 7 1759 1766 17400599 10.1093/brain/awm044

[B115] Pavese N Gerhard A Tai YF Ho AK Turkheimer F Barker RA Brooks DJ Piccini P Microglial activation correlates with severity in Huntington disease: a clinical and PET study Neurology 2006 66 11 1638 1643 16769933 10.1212/01.wnl.0000222734.56412.17

[B116] Sapp E Kegel KB Aronin N Hashikawa T Uchiyama Y Tohyama K Bhide PG Vonsattel JP DiFiglia M Early and progressive accumulation of reactive microglia in the Huntington disease brain J Neuropathol Exp Neurol 2001 60 2 161 172 11273004 10.1093/jnen/60.2.161

[B117] Cleren C Calingasan NY Chen J Beal MF Celastrol protects against MPTP- and 3-nitropropionic acid-induced neurotoxicity J Neurochem 2005 94 4 995 1004 16092942 10.1111/j.1471-4159.2005.03253.x

[B118] Norflus F Nanje A Gutekunst CA Shi G Cohen J Bejarano M Fox J Ferrante RJ Hersch SM Anti-inflammatory treatment with acetylsalicylate or rofecoxib is not neuroprotective in Huntington's disease transgenic mice Neurobiol Dis 2004 17 2 319 325 15474369 10.1016/j.nbd.2004.07.011

[B119] Sugars KL Rubinsztein DC Transcriptional abnormalities in Huntington disease Trends Genet 2003 19 5 233 238 12711212 10.1016/S0168-9525(03)00074-X

[B120] Khoshnan A Ko J Watkin EE Paige LA Reinhart PH Patterson PH Activation of the IkappaB kinase complex and nuclear factor-kappaB contributes to mutant huntingtin neurotoxicity J Neurosci 2004 24 37 7999 8008 15371500 10.1523/JNEUROSCI.2675-04.2004 PMC6729796

[B121] Henkel JS Engelhardt JI Siklos L Simpson EP Kim SH Pan T Goodman JC Siddique T Beers DR Appel SH Presence of dendritic cells, MCP-1, and activated microglia/macrophages in amyotrophic lateral sclerosis spinal cord tissue Ann Neurol 2004 55 2 221 235 14755726 10.1002/ana.10805

[B122] Kawamata T Akiyama H Yamada T McGeer PL Immunologic reactions in amyotrophic lateral sclerosis brain and spinal cord tissue Am J Pathol 1992 140 3 691 707 1347673 PMC1886170

[B123] Turner MR Cagnin A Turkheimer FE Miller CC Shaw CE Brooks DJ Leigh PN Banati RB Evidence of widespread cerebral microglial activation in amyotrophic lateral sclerosis: an [11C](R)-PK11195 positron emission tomography study Neurobiol Dis 2004 15 3 601 609 15056468 10.1016/j.nbd.2003.12.012

[B124] Henkel JS Beers DR Siklos L Appel SH The chemokine MCP-1 and the dendritic and myeloid cells it attracts are increased in the mSOD1 mouse model of ALS Mol Cell Neurosci 2006 31 3 427 437 16337133 10.1016/j.mcn.2005.10.016

[B125] McManus CM Liu JS Hahn MT Hua LL Brosnan CF Berman JW Lee SC Differential induction of chemokines in human microglia by type I and II interferons Glia 2000 29 3 273 280 10642753

[B126] Kuhle J Lindberg RL Regeniter A Mehling M Steck AJ Kappos L Czaplinski A Increased levels of inflammatory chemokines in amyotrophic lateral sclerosis Eur J Neurol 2009 16 6 771 774 19236470 10.1111/j.1468-1331.2009.02560.x

[B127] Keizman D Rogowski O Berliner S Ish-Shalom M Maimon N Nefussy B Artamonov I Drory VE Low-grade systemic inflammation in patients with amyotrophic lateral sclerosis Acta Neurol Scand 2009 119 6 383 389 18976328 10.1111/j.1600-0404.2008.01112.x

[B128] Rosen DR Siddique T Patterson D Figlewicz DA Sapp P Hentati A Donaldson D Goto J O'Regan JP Deng HX Mutations in Cu/Zn superoxide dismutase gene are associated with familial amyotrophic lateral sclerosis Nature 1993 362 6415 59 62 8446170 10.1038/362059a0

[B129] Clement AM Nguyen MD Roberts EA Garcia ML Boillee S Rule M McMahon AP Doucette W Siwek D Ferrante RJ Wild-type nonneuronal cells extend survival of SOD1 mutant motor neurons in ALS mice Science 2003 302 5642 113 117 14526083 10.1126/science.1086071

[B130] Lino MM Schneider C Caroni P Accumulation of SOD1 mutants in postnatal motoneurons does not cause motoneuron pathology or motoneuron disease J Neurosci 2002 22 12 4825 4832 12077179 10.1523/JNEUROSCI.22-12-04825.2002 PMC6757755

[B131] Pramatarova A Laganiere J Roussel J Brisebois K Rouleau GA Neuron-specific expression of mutant superoxide dismutase 1 in transgenic mice does not lead to motor impairment J Neurosci 2001 21 10 3369 3374 11331366 10.1523/JNEUROSCI.21-10-03369.2001 PMC6762496

[B132] Sargsyan SA Monk PN Shaw PJ Microglia as potential contributors to motor neuron injury in amyotrophic lateral sclerosis Glia 2005 51 4 241 253 15846792 10.1002/glia.20210

[B133] Beers DR Henkel JS Xiao Q Zhao W Wang J Yen AA Siklos L McKercher SR Appel SH Wild-type microglia extend survival in PU.1 knockout mice with familial amyotrophic lateral sclerosis Proc Natl Acad Sci USA 2006 103 43 16021 16026 17043238 10.1073/pnas.0607423103 PMC1613228

[B134] Boillee S Yamanaka K Lobsiger CS Copeland NG Jenkins NA Kassiotis G Kollias G Cleveland DW Onset and progression in inherited ALS determined by motor neurons and microglia Science 2006 312 5778 1389 1392 16741123 10.1126/science.1123511

[B135] Weydt P Yuen EC Ransom BR Moller T Increased cytotoxic potential of microglia from ALS-transgenic mice Glia 2004 48 2 179 182 15378658 10.1002/glia.20062

[B136] Ferri A Nencini M Battistini S Giannini F Siciliano G Casali C Damiano MG Ceroni M Chio A Rotilio G Activity of protein phosphatase calcineurin is decreased in sporadic and familial amyotrophic lateral sclerosispatients J Neurochem 2004 90 5 1237 1242 15312178 10.1111/j.1471-4159.2004.02588.x

[B137] Elliott JL Cytokine upregulation in a murine model of familial amyotrophic lateral sclerosis Brain Res Mol Brain Res 2001 95 1-2 172 178 11687290 10.1016/s0169-328x(01)00242-x

[B138] Yoshihara T Ishigaki S Yamamoto M Liang Y Niwa J Takeuchi H Doyu M Sobue G Differential expression of inflammation- and apoptosis-related genes in spinal cords of a mutant SOD1 transgenic mouse model of familial amyotrophic lateral sclerosis J Neurochem 2002 80 1 158 167 11796754 10.1046/j.0022-3042.2001.00683.x

[B139] Poloni M Facchetti D Mai R Micheli A Agnoletti L Francolini G Mora G Camana C Mazzini L Bachetti T Circulating levels of tumour necrosis factor-alpha and its soluble receptors are increased in the blood of patients with amyotrophic lateral sclerosis Neurosci Lett 2000 287 3 211 214 10863032 10.1016/s0304-3940(00)01177-0

[B140] West M Mhatre M Ceballos A Floyd RA Grammas P Gabbita SP Hamdheydari L Mai T Mou S Pye QN The arachidonic acid 5-lipoxygenase inhibitor nordihydroguaiaretic acid inhibits tumor necrosis factor alpha activation of microglia and extends survival of G93A-SOD1 transgenic mice J Neurochem 2004 91 1 133 143 15379894 10.1111/j.1471-4159.2004.02700.x

[B141] Tikka TM Vartiainen NE Goldsteins G Oja SS Andersen PM Marklund SL Koistinaho J Minocycline prevents neurotoxicity induced by cerebrospinal fluid from patients with motor neurone disease Brain 2002 125 Pt 4 722 731 11912107 10.1093/brain/awf068

[B142] Nguyen MD Julien JP Rivest S Induction of proinflammatory molecules in mice with amyotrophic lateral sclerosis: no requirement for proapoptotic interleukin-1beta in neurodegeneration Ann Neurol 2001 50 5 630 639 11706969 10.1002/ana.1256

